# Motivations for Industry Stakeholders in China, Vietnam, Thailand and Malaysia to Improve Livestock Welfare

**DOI:** 10.3390/ani9070416

**Published:** 2019-07-04

**Authors:** Michelle Sinclair, Zulkifli Idrus, Duong van Nhiem, Suporn Katawatin, Brendon Todd, Georgette Leah Burns, Clive J. C. Phillips

**Affiliations:** 1 Centre for Animal Welfare and Ethics, School of Veterinary Sciences, The University of Queensland, Gatton 4343, Australia; 2 Institute of Tropical Agriculture and Food Security, Universiti Putra Malaysia, Serdang 43400 UPM, Selangor, Malaysia; 3 Vietnam National University of Agriculture, Gialam, Hanoi 100000, Vietnam; 4 Department of Animal Science, Faculty of Agriculture, Khon Kaen University, Khon Kaen 40002, Thailand; 5 Seqwater, Process Documentation, Gold Coast 4211, Australia; 6 Environmental Futures Research Institute, School of Environment and Science, Griffith University, Brisbane 4000, Australia

**Keywords:** Asia, attitudes, culture, farming, motivation, intrinsic, extrinsic

## Abstract

**Simple Summary:**

Animal welfare is a global issue that is important to civilians in many countries. Despite this, large gaps exist between practices as recommended by a scientific understanding of farm animal welfare; the expectations and understanding of society at large; and the realities within livestock industries across the world. The reasons for this are as numerous as they are challenging to overcome, however, understanding what might motivate key stakeholders to make improvements could form a base from which to begin. The livestock industry are arguably the most important stakeholders with the capacity to make meaningful choices that impact the welfare of animals, yet seldom are they consulted as to why they might make choices that either improve or threaten the welfare of the animals. This study aimed to collect information about the motivations and barriers to improve animal welfare from leaders in the livestock industry. A complex relationship of motivators is uncovered, and the importance of factors such as financial benefit and food safety is discussed. Figures are presented to begin illustrating the relationship between motivators. The findings of this study serve to better understand the motivations of livestock stakeholders in these key Asian nations, and the barriers that may prevent them from making choices that improve the welfare of the animals.

**Abstract:**

Understanding what might motivate livestock stakeholders to improve animal welfare is useful information when developing initiatives that benefit from stakeholder engagement. This study was designed to assess the strength of motivating drivers in the development of attitudes to animal welfare, and the factors that impacted their ability to improve animal welfare. During a series of qualitative focus group sessions with livestock leaders across the same countries (Malaysia, China, Vietnam and Thailand), the current study presented livestock leaders (n = 139) with the most significant results in their country, and collected data pertaining to the meaning and applicability of these results. This data was then subject to thematic analysis to identify salient and repeated motivating factors and meanings. This process revealed a complex picture of relationships between motivators and the contexts that drive them. Figures are presented to begin illustrating these relationships. Some strong motivators were uncovered that were previously rated low in the survey (i.e., financial benefit) or not included at all (e.g., food safety). This paper also presents the opportunity to better understand the strength and relationship of extrinsic and intrinsic motivational forces behind animal welfare improvement.

## 1. Introduction

Animal protection, along with environmental protection and sustainable development are world social issues rated as being of the highest importance by citizens across vastly diverse cultures, from China, to Iran, to Norway [1]. Still, wide gaps exist between practices as recommended by a scientific understanding of animal welfare [2]; the expectations and understanding of society at large [3] and the realities within livestock industries across the world [4]. The reasons for this are as numerous as they are challenging to overcome, however, understanding what might motivate key stakeholders to make improvements could form a base from which to begin [5]. 

The study of motivation has been described as an inquiry into the factors that give impetus to action [6]. Many theories to understand human motivation exist [7]. Two of the most prominent are Maslow’s theory, which is based on a hierarchy of needs that begins with physiological needs and progresses to self-actualisation as each level is attained [8], and basic drive theory that focusses on instinctual need-driving behaviour through disturbances to homeostasis [9]. Other popular theories include expectancy–value theory, which ascertains that the best predictor of motivation (and therefore behaviour) is a mixture of expectation in an outcome and/or confidence to deliver an outcome, and the values and beliefs around the importance of the behaviour [10]. ‘Attribution theory’ also prominently appears in motivation theory, and proposes that people attempt to understand the behaviours of others by attributing intentions, beliefs and feelings to them [11]. How accurate the judgement of one individual regarding the motivations of another might be is unclear.

Although dominant theories in western countries, such as Maslow’s Hierarchy [12], point to the importance of understanding motivation in predicting human behaviour, they do not consider society in non-Anglo-Saxon cultures. When introducing cross-cultural dimensions such as ‘masculinity’, and ‘uncertainty avoidance’, as developed by Hofstede and colleagues [13], it is clear that assumptions cannot be made that the motivational forces are the same across regional border lines. It also becomes clear when reviewing the very significant literature on motivation theory that motivational factors vary not only with country, but also by situation, role and individual.

One way to understand motivations in the context of agricultural animal welfare is to ask livestock industry professionals themselves, in order to find themes that can be applied across this specific sub-set of the population. Understanding what motivates stakeholders to act may assist in engaging them in animal welfare initiatives [14].

The need for engagement and isolation of a ‘mutual benefit’ for animal industry stakeholders is key to successful international animal welfare initiatives [15]. In terms of animal agriculture, no region is more presently significant than Asia. China alone is responsible for nearly 40% of the global animal agriculture output [16], along with being the biggest producer of fish, pig and duck; and second biggest producer of chicken after the United States [17]. Further to that, China and India host more consumers than the rest of the world combined, with 1.4 billion and 1.3 billion respectively [18]. As an extremely important region for animal agriculture, a region diverse with sub-cultures and a region often poorly understood by the Western world, the need for relevant and practical information that can underpin the animal welfare movement in Asia is great. When considering the international relevance of animal welfare and protection, both as a growing social interest [2] and from the perspective of global trade relations [19,20], it is important to acknowledge the major impact culture plays on attitudes and practices. National culture is the most significant demographic influencing animal welfare attitude [15,21,22], and therefore an understanding of perspective by country is essential for effecting positive change. In addition to their individual economic importance in the agriculture sector in Australasia, the countries chosen within the study also serve to highlight key cultural differences within the Asian region. For example, while China, Vietnam, Thailand and Malaysia all share the common Asian cultural characteristic of being highly collectivist nations (focused on the interests of the ‘we’, or group, rather than the individual, or ‘I’), along with other similar trends, the countries still substantially differ from each other in terms of general culture and way of life [13]. While Vietnam shares some geopolitical history with China, Vietnamese culture tends to be far less long-term orientated than that of China; despite the strong Chinese influence in Malaysia (including a quarter of Malaysians identifying as Chinese), Malaysians are far more likely to engage in indulgence than Chinese nationals, and the Thai are far less likely to prefer and find comfort in hierarchy than their immediate neighbours in Malaysia [23,24].

A background study, conducted in 2017, investigated motivators of Asian livestock holders in the context of animal welfare in a questionnaire of over 1000 livestock industry stakeholders in China, Malaysia, Thailand and Vietnam [25]. This research found a significant correlation between country and the strength of different motivating forces. For example, the strength of religion as a motivator was particularly strong in Malaysia, where 62% of the population are Muslim [26,27]; and the strength of ‘peer opinion’ was particularly strong in community-based Thailand. Motivators such as ‘law’, ‘personal knowledge’ and ‘company approval’ were significantly important in all the countries surveyed. While this study can be considered to have illuminated a novel area of research, the quantitative nature of the survey did not provide information into what respondents were thinking, and why they ranked the motivators in the way they did. Advised by the significant results of this earlier study, the current qualitative study was undertaken with the aim of developing a deeper understanding of the positions and attitudes of stakeholders. Specifically, we aimed to gain insight into motivational forces for improving animal welfare by country; and to understand how the motivators interact with each other. Further information gained through the current study aims to understand the deeper motivations of livestock stakeholders towards animal welfare in an applicable way that will be useful in the development of international animal welfare initiatives, therefore increasing ability to engage livestock stakeholders in animal welfare improvements. Results are presented by key motivators, under which key themes have been identified and quantified; further qualitatively supported by participant quotes that best illustrate the attitudes and opinions that are represented in the data.

## 2. Method

Eleven focus group sessions were held in Vietnam, Malaysia, Thailand and China (see Table 1). These countries were chosen due to their diverse cultures within the Asian region, the scale and importance of their agriculture industries, in particular those of importance to Australia (the country in which this study originates). Examples of this include the size of industry in China (pigs, chickens and fish in particular), the scale of the chicken product trade in Thailand, and the live trade (particularly cattle) relationships Australia has historically held with both Vietnam and Malaysia. These countries also presented ideal focus within this study, given the previous data collected for these countries, and therefore the ability to conduct more targeted research. Locations were geographically dispersed locations in different areas of each country (i.e., south, north, central, capital and regional), in an effort to capture potentially varied sentiments between domestic regions. Advised by an earlier survey conducted with livestock industry stakeholders [25], industry leaders were invited as representatives of the livestock industry to discuss the significant motivators in a semi-structured format.

Participants were invited through country-based collaborators utilising selection criteria that included: they were leaders in the agricultural sector, working for an organisation with a maximum of five government vets, and have the ability to implement change into private businesses. The majority were private industry leaders (e.g., pig or poultry slaughterhouse or production managers or owners). In some focus groups, certain participants were known to each other as professional colleagues.

Although plans were made for five to seven participants in each session, the actual number of participants present on the day varied from three to 14, due to cancellations at the lower extreme and heightened interest at the other. Sessions with more participants often ran past the scheduled 3.5 h in order to allow all participants adequate opportunity to contribute.

After presentation of the five highest ranked motivators and factors impacting on ability to improve animal welfare (Table 2)—that had been determined in a previous study [25]—stakeholders were invited, as participants, to discuss these results. The lead researcher facilitated all groups by prompting participants to discuss the following: their level of agreement with the results, what survey respondents may have specifically meant, the reasons they may have for answering in this way, and any other thoughts regarding the results they wish to share. This activity comprised approximately one third of the total focus group content during each session, lasting approximately 1.25 hours depending on location. The remainder of the content focussed on specific animal welfare issues, benefits to improving welfare and solutions, and willingness to embrace pre-slaughter stunning, which will be presented in subsequent manuscripts. 

Dialogue was voice recorded during the sessions and additional field notes were taken by a research assistant. Both data sets were used to create abridged transcripts of each session. As participation was subject to verbal translation during the sessions in most instances (except for some Malaysian and Thai participants who spoke English), verbatim transcripts were not possible.

Transcripts were uploaded into NVivo software (QSR International, Melbourne, Australia) for Mac 11.4.3 for analysis.

## 3. Analysis

Thematic analysis was utilised, in line with a Grounded Theory approach. The top five motivators were classified as themes and coded as nodes in NVivo. Data was then divided into relevant logical sub-themes where they were present, and identified both through manual familiarisation with the data, and using software tools such as ‘word frequency’ within each theme. Additional themes (motivators) were identified along with those presented to the participants and are presented in results as ‘other: financial benefit’, and ‘other: food safety’. At the completion of analysis and coding of themes and sub-themes, no new themes emerged from the data, suggesting data saturation. The same lead researcher that conducted the focus groups also coded all themes/nodes and conducted the analysis.

The initial intention during the focus group sessions was to separate responses to ‘*encouragement* to improve animal welfare’, and ‘*ability* to improve animal welfare’. However, the difference between these two concepts may not have been clearly comprehended by the participants during translation in the focus groups. Instead, when the theme was presented in the context of ‘encouragement’ or ‘ability’ this was noted in the results.

To avoid presenting misleading data, linguistics and tone are not reported, as all data was translated, abbreviated, and summarised through verbal translators during the sessions, from four different languages to English. For this reason, rather than focussing on word usage, more attention was paid to careful analysis of the key themes (motivators), the frequency of their appearance across countries, the general context and interpretation of their meanings, and their interrelationships. However, word frequency functions in NVivo were still utilised in identification of sub-themes and are reported accordingly in results.

This study was granted human ethics approval by the University of Queensland Ethics Committee, approval number: 2017000628.

## 4. Results and Discussion

The following section presents the results of the data and discussion in sections dedicated to each key motivator as measured in the study and discusses additional motivators that were persistently raised as important in multiple sessions. In relation to each motivator, identified key themes are quantified, summarised (see Table 3), and participant quotes are presented to better illustrate the attitudes and opinions that were collected in the data.

The 11 focus group sessions included a total of 83 participants spread fairly evenly across the four countries: 20 from Vietnam, 21 from Malaysia, 19 from Thailand and 23 from China (see Table 4). Across the countries the participants comprised 15 veterinarians, 40 business representatives from animal industries, 17 government representatives and 11 senior agricultural academics, such as professors and lecturers.

## 5. Motivators

### 5.1. Law

The participants reported wide agreement with the importance of law as an extrinsic motivator, with respondents in all countries (11/11) agreeing with its highest ranking as a motivator to improve animal welfare standards. However, the position of law as the sole highest motivator is also dependent on enforcement, the ability to draw from prescriptive standards to meet legal requirements with knowledge and is closely related to that of company approval (discussed below). Indeed, some participants referred to law when they meant corporate policy (particularly in China). 

Contextually, the participants mostly referred to farming laws (‘farm’ being the most common associated word, secondary to ‘law’ and ‘animal’) in this theme. All sources contain the notion of ‘need’ in relation to law, and the power of the law was a sentiment that was shared across participants in all countries.

### 5.2. Need for Standards

‘Standards’ was the most common word within statements concerning animal welfare law, and mostly in relation to the need to have formalised standards in existence, in addition to law. In places, ‘standards’ was used interchangeably with ‘law’. ‘Standards’ were also discussed from government mandate and company driven perspectives. Where standards were discussed as ‘needed’, in addition to the law, it was for their prescriptive utility, making available clear direction. ‘So far only one article in Vietnam (Legal Article 21) exists, and there is not much detail in the description’ <V_HN>, ‘law should be more detailed and better, we need more guidelines and documentation, procedure and protocols’<V_HC>.

After introducing a ‘baseline’ of law, standards (sometimes company led) were viewed as the conduit to implementing the law in a practical way, ‘The government issues laws, but that law is only the baseline’ <C_BJ>. While some suggest that companies themselves are in the best position to create workable standards (discussed in more detail in ‘company size and structure’ below), many considered that standards should be government devised and generic, ‘a set of farming and slaughtering standards should be made’ <C_GZ>. To many participants, government was in the best position to create these standards, and industry was in the best position to lead the implementation; ‘As I am an animal farmer and livestock producer, I think we should apply and follow all guidelines of the competent authorities about animal welfare, and we as farmers, we should be the leaders in this area’ <V_BT>. 

Participants of one session in China, and one session in Thailand considered that standards and laws that were specifically locally devised, researched, and relevant to the local region were necessary. On recounting a recent conference speech delivered by the Agriculture Minister for China, one participant stated ‘we cannot just have standards towards animal welfare that are not realistic in the Chinese situation, they must be based on our own situation, and not the situation of other countries’ <C_BJ>.

Lastly, standards were also discussed in a frustrated context of not knowing which standards to follow, particularly in regard to export where the laws and standards of multiple countries may apply ‘we have talked already about many different kinds of law [...] different countries will be slightly different, so which law should we follow?’ <T_BK). When asked if international standards such as those developed by the World Organisation for Animal Health (OIE) may be of use if applied in these situations, it was remarked that ‘no, because this is the standard guideline, but not everyone around the world accepts it [...] some say ok, some say not ok [...] even if OIE or Europe (deem a practice acceptable) then Americans say not ok [...] then Americans say a practice is ok, then Europe say it’s not ok’ <T_BK>. 

### 5.3. Company Size and Structure

‘Company’ was the second most used word within the data concerning animal welfare law, specifically in relation to the factors that impact on the efficacy of the law and the strength of law as a motivator. While ‘company approval’ was amongst the top motivators to improve animal welfare (discussed in ‘company approval’ below), it is unsurprising that the role of ‘company’ appeared so frequently in relation to law. Many participants saw an opportunity for companies to drive higher standards. It was suggested by one participant, with fellow participant support, that company standards could be much higher. ‘In my company I think that the workplace has a higher order than law. If standard of the workplace is stronger, then the workplace policy and procedures will be stronger than the law’ <T_CM>. The power to implement standards within industry were viewed as particularly strong with bigger companies. A focus group participant from a large poultry production company stated, ‘as a contract farm, they should know that if they are going to set up the farm there are some standard protocols they have to follow. As a big company, they have to look after that and tell them more, but they know that, and they will follow those protocols if given by business [...] as a farm they have a standard, they have to follow them’<T_BK>. This sentiment was shared in most focus groups, with stakeholders drawing attention to a large disparity between the impact of law between larger and smaller scale businesses, with some sharing that larger businesses may be in a position to motivate improved standards by setting protocols/standards and enforcing them throughout the supply chain.

Regardless of commercial size and structure, participants believed law was the ultimate extrinsic motivator, particularly in the face of animal welfare improvements that are not perceived to yield substantial economic return. ‘It’s important to level the playing field, otherwise someone is doing it and the other people can’t survive, the law is fair as everyone is doing the same thing’ <M_NS>. ‘Everyone is (the) same, when you talk about business (it) must be profit [...] should be win–win but doing good things for the animals should not be bad for business, so if that is the scenario, then law must be set in’ <M_NS>.

### 5.4. Awareness of Law

In almost half of the sessions, participants drew attention to awareness of the law, as a caveat on the strength of law as a motivator. They suggested that stakeholders need to know what the technical standards are and how to essentially comply with the law, but also that laws exist (Thailand, Vietnam and Malaysia). It is suggested that the knowledge of the simple existence of a law (which is thought to be lacking in many cases), may be enough to motivate a workforce to take animal welfare more seriously. ‘So, first law and monitoring can influence the techniques and knowledge and these can influence the common people/farmers and this will lead to the improving of a person’s knowledge and then to of improving animal welfare’ <C_BJ>, ‘maybe law can be used as a way to communicate.’ <C_BJ>, and, when it comes to enforcement, ‘I ask the police for procedures under the law and that encourages my staff’ <V_HCM>.

When speaking of awareness of the law as a motivator to improve animal welfare, participants suggested that government bodies should be responsible at all levels, ‘law at different levels [...] law and regulation should be spread more and more widely, and governments should be involved at all levels’ <V_BT>. Furthermore, creating a law was not sufficient in itself, ‘government has (the) responsibility to make law and also publicise and make everyone know about the law’ <C_BJ>. Discussion again included the relationship between size of operation in relation to likelihood of law awareness, ‘I receive documents including information on law from vet authorities and local government, I am running intensive farms and so find out and know legal and technical documents, but I don’t think the other small-scale farmers get the same information’ <V_HN>.

### 5.5. Power and Impact of Law

When discussing the motivational value of animal welfare law, the power and impact of the law was discussed in every session in some way, mostly in regard to agreement in the highest strength of law as a motivator, but also in regard to some of the gaps where the efficacy of law as a motivator may be jeopardised. In regard to the power of the law, comments such as, ‘laws control society and (the) community’ <V_BT>, ‘laws control society’ <V_HN>, ‘all activities in society are controlled by law, law governs everything’ <V_HC>, ‘everyone agrees law should be top, everyone’s afraid of the law’ <M_KL>, were commonplace in all groups, particularly in Vietnam. ‘Because most people have low awareness and knowledge of animal welfare, we need law; attitude is very poor’ <V_HN>.

In addition to a complete absence of law or a lack of awareness of the existence of laws across the countries, the biggest perceived vulnerability in the efficacy of law was in its enforceability. ‘We need law, and when we need law we need enforcement; local government and police are very important’ <V_BT>, ‘If it’s not necessary people won’t do it, because for business rules and regulations, for every change we make, (it) accrues more costs, but as for profit coming out, who can guarantee that? It’s always profit and loss [...] so unless law is enforced, they are less inclined to make changes’ <M_KL>. When discussing the broad and less prescriptive nature of Vietnam’s first animal welfare law, Article 21, one participant stated ‘also we have law, but a very common problem in developing countries is enforcement of law is very difficult. Breaches are only criticised, not penalised or punished, it’s not serious or strict. Some farmers violate the law and they should be penalised, but they (the authorities) aren’t strict with them’ <V_HN>. In Malaysia, animal welfare laws have been gazetted, but are not necessarily enforced at the time of writing of this paper, awaiting the production of prescriptive standards that may be enforceable. Standards may also address gaps in the efficacy of law in cases where more detail is needed, or an operation is not covered by law; ‘law for poultry only applies if you have more than 500, otherwise (it’s) not binding’ <M_KL>.

While the strength of established and enforced law seemed reinforced in all groups, some discussed the difficulty of getting to the stage of making law. This sentiment was particularly prevalent in the China, Zhengzhou focus group <C_ZZ>.

Commenting on the requirements of any new law, one participant stated that ‘the issuing of the law should be (firstly) based on what society wants, second the country has the need to trade with other countries so that needs to be considered, and thirdly public demand; if public demands there will be law’ <C_BJ>. 

The strength of law as a perceived motivator for increasing animal welfare was shared across countries in the initial study and this was, not surprisingly, echoed in the current study. The close relationship between law and company approval, and the role law plays when a perceived lack of financial benefit exists, did not emerge in the first study. The challenges faced by relying on the law as a motivator included lack of enforcement, lack of standards, gaps in law, lack of prescription in law, and the difficulty of passing law where it is not already enacted. Although the law was agreed to be the most important motivator in most cases (except where there may be financial benefit), when other motivators mimic law and become an external pressure or obligation, such as religion or company policy, their power begins to equal or even rival that of law. The degree to which this occurs may be most impacted by the seniority of the stakeholder within the industry and company. From the results, law emerged as an external fail safe for animal welfare improvement, in the absence of perceived value, and knowledge.

## 6. Workplace Approval

Approval of the company, or workplace, to make animal welfare improvements appeared throughout the data as an extrinsic mitigator of ability. That is, participants generally discussed workplace approval in the context of other motivators being potentially more important to drive the improvements; however, without workplace approval the improvements became impossible. ‘Company approval relationship; law is the most important motivator, but company approval is the most practical’ <T_KK>. ‘I think even though we have law, the enforcement and monitoring bodies (police) cannot go in to inspect every day [...] in my opinion I think company approval is more important, as if the company agree or allow to do that, they can monitor it every day, so (it’s) better than law’ <T_KK>.

The strength of workplace approval when it comes to stakeholders perceived ability to improve welfare is likely to vary depending on the role the stakeholder holds within the industry, and how senior or able to enact change that stakeholder is. ‘If you are working directly with animals, then your work depends entirely on what your manager says’ <T_KK>.

### How to Encourage a Workplace to Give Approval for Improved Animal Welfare?

The question was posed in each group, ‘how then do we motivate companies to give their approval?’, and the answers saw little variation between countries. The answer unwaveringly focused on profit. ‘As a company they are doing business, if you want the company to approve, they have to get some benefits back [...] even though they know this is good for animal welfare, if it doesn’t gain back money, they don’t do it’ <T_BK>. ‘Companies want to make money, so when animal welfare can improve their benefits, they will incorporate that notion’ <C_BJ>. ‘As the perspective of the company, they will stand at point of improving profits so they will consider if pig feel cold, they use warmth and when they feel hot, they will use air con or fans to reduce temperature, so they stand at point of improving profits first’ <M_ZZ>. ‘I think cost/benefit is more important [...] so (they are) more likely to improve if (there is) financial benefit’ <M_KL>. 

Some participants drew attention to the nature of the relationship between law and company approval as a source of encouragement. When summarising discussion with the Malaysia, Kuala Lumpur Selangor focus group, it was asked by the facilitator if participants believed that companies would only improve animal welfare if required to by law, or if assured of financial benefit, and there was participant consensus. In China, the relationship was again explored. ‘From the company perspective, it is firstly about the efficiency and economic benefits, if certain law can be issued to regulate then yes, they must do it, but from a very shallow level; not from their heart. But if they find out there is benefit, they will do it for themselves’ <C_GZ>’. This returns to the need for knowledge around the benefits of improving animal welfare (discussed above in ‘personal knowledge’). ‘Company needs to know the benefits to the company [...] the company will need to know the advantage of doing animal welfare, and under the advantage can be benefits either in terms of financial benefit, or that it’s better for farmers’ <T_KK>.

Companies, once engaged and giving their approval to changes, are believed to be in a position to drive animal welfare standards higher than the law. ‘In my company I think that the workplace has a higher order than law [...] the standard of the workplace is stronger, as the workplace policy and procedures will be stronger than the law [...] if the law says you need to do these *three* things, the workplace policy will be *four* or *five* things’ <T_CM>. ‘To set up the policy you base it on law, then make it better than what the law wants; example of space [...] if law says 0.4 the company policy will be more than 0.4 to 0.5′ <T_CM>. The China, Zhengzhou focus group believed that this may be less so for China, where confusion around the differentiation between law/government and company policy exists. This may be tied to the close relationship the Chinese government has with businesses in China, the influence of the law in China, and business risks associated with deviating from legalised standards.

## 7. Personal Knowledge

Personal knowledge was frequently discussed in the context of being both an intrinsic motivator and a barrier. In each session, statements were made which indicated that relevant general knowledge about animals’ needs was at a low level, and where knowledge was improved in certain areas, it was likely to result in a desire to improve animal welfare. ‘Animal welfare is very new, even among vet officers, very few have much knowledge at all. Even with vet authorities, it takes a very long time to spread (the) concept to lower levels’ <V_HN>. However, there was often perceived to be a relationship between knowledge and standards, in that it was commonly believed that a ‘lack of knowledge’ was directly linked to lower standards of animal welfare. Further analysis to identify subthemes was conducted, to ascertain what knowledge was considered lacking. In regard to who required the knowledge, participants mostly referred to industry stakeholders (56%) and the public (39%), frequently focussed on children, but also commonly government bodies (5%). The following sub-sections present key sub-themes found in the data concerning personal knowledge. In this case, they present the nature of personal knowledge that is needed, to aid animal welfare improvement.

### 7.1. Stakeholder Technical Ability

When prompted to discuss what personal knowledge was needed, most emphasis was placed on industry stakeholders (56%), specifically focussed on technical ability to improve animal welfare, but inclusive of general knowledge of animal welfare, law (discussed above), and awareness of the benefits for addressing animal welfare (both discussed below). ‘Training is the first priority for stakeholders who directly work with animals’ <V_HC>. ‘To improve animal welfare, I think we need to educate farmers on what is animal welfare and how to do it’ <T_KK>. 

Such education includes improved knowledge of animal-welfare-friendly practices, and how to use available tools and equipment. ‘I think that the loading process is quite important and depends on the workers; if they have good experience and know about the behaviour of animals they can do it easily and the animal will feel better, they won’t suffer from (the) loading process [...] however if the worker has no skill [...] (he, she) will use rough handling or use electric goads <T_CM>. ‘Stakeholders need knowledge of how to achieve the aim of animal welfare [...] how to do animal welfare [...] like one example is for sows: they use the pens in which they cannot move, but now they make the space larger according to the size of the sows’ <C_ZZ>. ‘Emphasis is more on the right procedure [...] has to be clearer and be informed to the workers, owner [...] so they know exactly how to do it <T_CM>. 

In addition to the correct management of animal welfare friendly protocols and use of tools and equipment, participants also spoke of a need to improve handling skills, often tied to the motivator of ‘personal value’ (discussed further in ‘link to personal value’ and ‘personal value’ below), and in particular where the associated value of animal welfare might be reduced. ‘Workers maybe come from a different awareness and different background and in the beginning, they are probably quite rough with the animals [...] so we need to educate them and teach them to think more about animals’ feelings and become more gentle’ <T_CM>. ‘The way I teach farmers is I tell him to use himself, and encourage the feeling of the farmers to consider “in that condition how do you feel? [...] if you feel good, the animals will feel good also [...] if you feel not so comfortable, the animal will probably not feel so good either [...] so if you feel too hot, the animal will feel too hot also”’ <T_CM>. Thus, some participants advocated that an anthropomorphic approach to evaluating animal welfare may be useful for stakeholders.

Participants also suggested that training of stakeholders could impact on the value attributed to animal welfare and improved processes; ‘at the beginning we need to closely supervise them and then after that they will learn and it will become normal practice for them [...] it had probably not entered into their perspective [...] but if you tell them to do (something) and closely supervise every day, it will become inside that person, and after that, we won’t need to monitor closely anymore’ <T_BK>. In addition, knowledge of the animals and their behaviour was also raised as an area requiring improvement, ‘first we need to understand the animals so we can improve their (the person’s) behaviour’ <C_BJ>.

One group in Thailand expressed frustration that despite seeking animal welfare knowledge, it was not clear what farming and slaughter systems were recommended. ‘What is the best for slaughter of chickens? In the beginning they say electrical or gas stunning, now they say atmospheric; so how will this end [...] once we implement it costs millions and millions, then once we implement someone says, “oh this (is) not welfare”. We have experience buying a machine from France and they sent a certificate saying (it) is approved by animal welfare [...] then the British people come and say no [...] so we bought the machine and haven’t even used (it) and we had to buy another one [...] and then we get confused’ <T_BK>.

Finally, the remark was made on five occasions that government, or ‘competent authorities’ also require an increase in animal welfare knowledge. ‘I think the problem is the government section that don’t know much about animal welfare [...] this is quite a problem, because under law this government officer is the one who is monitoring and enforcing, but they don’t know much about welfare’ <T_CM>.

### 7.2. Public General Knowledge

Although the statements regarding knowledge were most often directed towards industry stakeholders, a larger emphasis than expected was also placed on the general knowledge of the public. The public general knowledge that was expressed as being needed was centred on animal agriculture, and animal welfare. This suggests that the ranking of ‘personal knowledge’ as a motivator by the stakeholder respondents in the original questionnaire study may have at least in part been in reference to the knowledge of those around them (peers and public), rather than their own. ‘Stakeholder knowledge is more important, but the general knowledge of the public should improve too’ <V_BMT>. ‘Everyone needs the knowledge; stakeholders and (the) general public’ <V_HCM>. This sentiment was often clarified to be in regard to consumers, but also in the context of general animal welfare value building within a society that will probably be influencing stakeholders’ base values and knowledge. In particular, it will be influencing peoples’ expectations of what may be acceptable treatment of the animals before entering the industry.

Children, rather than consumers in general, were the main subset of the general public deemed most in need of animal welfare education. This was consistent across the four countries, as participants believed that children were an investment in the future. ‘All children should be educated for animal welfare knowledge and then as they grow up it grows [...] it is not lack of care, just the knowledge is poor[...] stakeholders, farm owners, slaughterhouse owners, slaughterhouse workers; children are the future (and) education for children is for the future [...] specific education for stakeholders is for short term’ <V_BMT>. 

The knowledge required for the children can be classified into three categories; basic knowledge about the animal species, what animal welfare is, and to value the life with empathy (with the exception of Thailand, where Buddhist roots place great focus on empathy for life). ‘the public doesn’t really know what’s going on [...] some children in Singapore don’t even know where milk comes from [...] (my) cousin’s child sees a cow when he visits at KL and says, “wow look at the dog”’ <M_KL>. ‘You’d be surprised, in a room of professionals, engineers for example, they still ask, “how can a hen lay eggs, when no mate/partner?” [...] those questions are being asked by intelligent people, knowledge is very low’ <M_KL>. ‘Children also need to be taught about agriculture, where eggs and milk come from for example, doctors, accountants, lawyers [...] (they have) no idea about where anything comes from [...] then the next step is to talk about animal welfare. Then you don’t have to force them through law’ <M_KL>. When one group in Malaysia was asked if this general animal-based knowledge would make it easier to then discuss animal welfare with industry stakeholders, the participants were unanimous in agreement.

### 7.3. Knowledge of Benefits

The third area of personal knowledge believed to be lacking, was that related to the benefits of improving animal welfare. ‘I think, train and tell workers at my farms what is animal welfare and what are the benefits of animal welfare [...] if you provide good conditions to animals it will improve the productivity and quality of products’ <V_BT>. ‘Who should be responsible for promoting the notion of animal welfare first, is industry stakeholders, (they) need to maximise their benefits, so they (advocates) need to make the benefits known to them’ <C_BJ>. ‘The people should know what the benefits are’ <V_HN>. ‘Benefits you can get from animal wellness [...] educate all stakeholders’ <M_NS>. ‘Everyone is (the) same, when you talk about business (it) must be profit [...] so (it) must be education about business benefits’ <M_KL>. 

In relation to knowledge of benefits, a sentiment of ambiguity regarding financial benefit to improving animal welfare was present in some groups, in particular in China (discussed in ‘financial benefits’ below), and in some cases, a caution for consumers; ‘If you want to educate children about buying premium product, we can tell them the benefits but (you) must weigh up with (the) cost of living [...] at this point the benefits are negligible’ <M_NS>.

### 7.4. Link with Personal Values

As is the case throughout this dataset, none of the assessed motivation stands alone, yet rather forms a part of a broader picture. This is the case with personal knowledge, and a link to personal values. In many cases, but in Thailand specifically, it was believed that personal values towards animals existed (regarding a value for their life and reduction of suffering), but the required knowledge to deliver improved animal welfare was often lacking. ‘As an example, it’s quite common for Thai people, when they see stray dogs, they try to give them some food or take to their house, but they think that if you offer them food and water it’s good enough, but the welfare of the dogs on the street is probably not great’<T_BK>. The presence of statements linking the two in the data suggested a connection between ‘knowledge’ and ‘personal values’ and suggested that they can influence each other (particularly knowledge influencing values), and that one of these motivators without the other may lose overall motivational efficacy. ‘I think for slaughterhouse or abattoir (values) perspective, (it) depends on the person’s knowledge and awareness <V_HC>.

## 8. Tools and Resources 

Most of the data coded to the theme tools and resources was related firstly to knowledge (as above); that is, knowledge of which tools and resources to use specifically, and then how to use these tools and resources. Secondly, often prompted by the researcher, participants discussed what they believed survey respondents meant by ‘tools and resources’.

The China, Beijing focus group session discussed ‘tools and resources’ as referring to ‘scientific information’, while other groups tended to focus on physical aspects, specifically farming husbandry equipment for low stress handling such as ‘pig boards and noise makers’ <V_NH>, and equipment for pre-slaughter stunning. ‘Some specific tools that are needed in slaughterhouses are good lairage, handling equipment and stunning equipment’ <V_HN>. ‘Stunning, if it’s a big company, it’s no problem as they already have all the equipment, but if small farmers, they don’t have the stunning equipment’ <T_CM>. In Vietnam, participants discussed different province regulations about stunning equipment, which is legally prohibited in many places. ‘Stun gun (captive bolt) is legal in some areas, but people think it is a weapon [...] they think it’s a gun only for military and police [...] my company still uses stunning equipment, but it’s difficult as sometimes I have had a problem with the use of the cartridge [...] the problem is with explosives or mortar’ <V_HN>.

Participants also commonly drew reference to physical space as a resource that impacted their ability to improve animal welfare. ‘The tools are mainly in reference to facilities provided by pig farms, like the size of pens’ <C_ZZ>. ‘If the farm is small, they cram them in more [...] so the size of the farm impacts ability’ <M_KL>.

In addition to the physical tools and resources, two groups discussed the lack of staff as a missing resource, and the lack of money to make improvements. ‘Many things are needed but mainly enough money and human resources [...] enough people’ <V_HN>. ‘Resources also refers to the money’ <M_NS>.

## 9. Personal Value 

**Figure 1:**
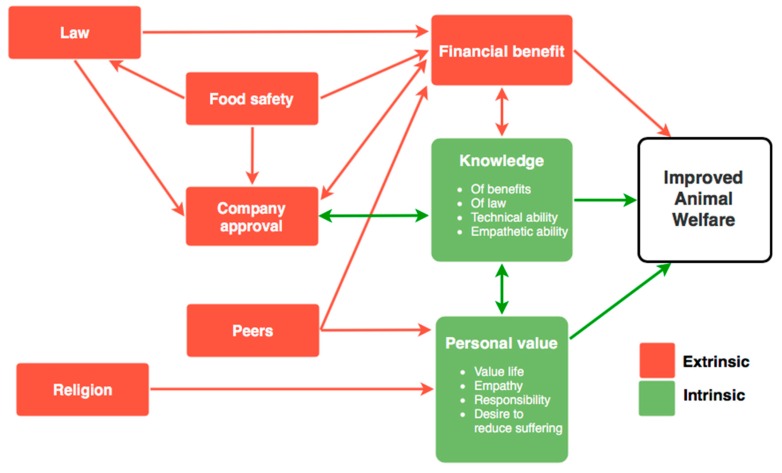
The relationship of intrinsic and extrinsic stakeholder motivators on improving animal welfare in the Asian livestock industry.

Personal value as a motivator was intended in the initial questionnaire to be in reference to intrinsic value of animal life and is presented as such in this section. However, the term has been broadened as a result of this study, in light of the understanding of stakeholder participants, to also include extrinsic value, and is presented as such in Figure 1.

Personal value as a motivator was particularly relevant in Thailand, with initial questionnaire results suggesting it was the single most important motivator for the Thai respondents in considering improving animal welfare (see Table 2). Personal value was tied to a need for knowledge (as discussed above), and the results suggested that intrinsic value was inherent in many Thai farmers. ‘[...] all or most of Thai farmers already have their personal value towards animals, however if you’re going to make it more, I think if we educate farmers to know if they do animal welfare, it will increase the benefit for the farmers, and then they will feel it’s a must to do that’ <T_KK>. ‘They think that as farmers they don’t know what animal welfare is, in terms of definition or phrase, but when we take care of animals, we want our animals to be comfortable; to be healthy. And all these farmers will do these kinds of things to make sure their animals are well. Some things that they do will be collectively good for animal welfare, but they don’t know it as such as they don’t know the phrase. Some things they do by good intention, though may not be good for animal welfare’ <T_BK>. ‘As Thai people we are so kind especially for the animals that we take care of by ourselves. We will be more gentle to those kinds of animals. Because of our kindness, our culture and related to our religion (Buddhism). Even when he goes to do a service for the farmers to give injections, he (veterinarian) has to be very kind, or the owners are not happy’ <T_CM>. ‘Thai people take personal value as the most important’ <T_KK>. ‘Thai people are very kind to the animals and have a very close relationship with the animals we take care of; and some religion is involved in this; mainly Buddhism’ <T_KK>. The value of animal life is not an outward or conscious effort or motivator in this case, it simply exists as a cultural value or norm. This was attributed to roots in Buddhism for Thailand (discussed in ‘religion’ below), ‘I think personal value may be related to our Buddhism as, as a Buddhist, first rule is be kind to animals, to the living things; even though we are in the business of animal production we still try to be so kind’ <T_BK>. In the survey, ‘religion’ was rated low as a motivator in Thailand, which could indicate a lack of belief in higher power; however, through this study it became clear that a spiritual belief is paramount as a motivator in animal welfare. Because Buddhism is viewed as a ’philosophy’, or ‘way of life’ intrinsic to Thai Buddhists, it was not apparent when Thai stakeholders responded to the motivational strength of ‘religion’, which they see as extrinsic, and not in line with their Buddhist beliefs. It is clear through this study that the strength of spirituality in Thai Buddhism was classified within the survey as strength of ‘value’. ‘If you say it is related to religion, the religion is like an external law, to tell you to do this and that [...] but it also influences us, as we have grown up under the Buddhist ideals, and somehow (it) is like inside yourself [...] that’s why they choose personal value, not religious’ <T_BK>.

This influence seems to have crossed the border into some areas of Vietnam; ‘most people affected by Buddhism believe if you do bad things to animals and other animals you have bad karma’ <V_BT>.

The data showed that value was of particular motivation in Thailand; however, participants in all countries (10/11 sessions) made statements to suggest that personal value was an important motivator to animal welfare improvement even though they were not specifically questioned about it. In Malaysia, it was noted that value was tied to religion (mostly Islam), and that the associated value of the life of species was directly related to religion (discussed further in ‘religion’ below). It was believed that for this reason religion had been rated the highest motivator in Malaysia, rather than ‘value’. ‘Because of (the) beliefs and values people hold personally for livestock, it will affect what stand they take; we are generally religious people’ <M_KL>. When discussing what knowledge to share to improve animal welfare in Malaysia, one participant with wide support from the remainder of the group stated that they need to ‘realise that animals are creatures of God, created by God, and we should appreciate them [...] it is tied with religion and social values’ <M_KL>.

In some sessions, participants noted that the value associated with animal life (and therefore the motivational strength to improve animal welfare) was likely to vary not only in cultural contexts but also in the context of different species. ‘I have observed that the tendency of people is to be more and more caring about dogs and cats, and if we can do something to encourage people to expand that attitude from domestic to other animals, from pack animals to farm animals, it would be better’ <V_HN>. ‘I think it is different between the companion animals and livestock animals, the concerns about animal welfare will be different because of the close relationship between companion animals and humans’ <T_KK>.

Some supporting statements were made in relation to the general societal improvement of the associated value of animals and their welfare; ‘of course now we can see personal values among public are getting higher, particularly with social media for example, things get full support from people, so when you look at the general public we may see awareness increasing’ <M_NS>, however the caveat of financial motivator (discussed below in ‘financial benefits’) frequently ended these statements; ‘but when we talk about implementing animal welfare and incurring costs, they become more and more reluctant because they need to fork out something [...] if you ask them who supports (animal welfare), they all do, but when you talk about implementing/committing, it’s not easy’ <M_NS>. ‘I still agree with (the motivational strength of) personal values, I believe they already have that, but also there’re other factors that may influence personal value, like money income’ <T_KK>.

## 10. Religion

Although religion was briefly discussed in the Thailand sessions, in the context of Buddhism being a ‘personal value’ rather than ‘religion’, it was only otherwise discussed in Malaysia, where ‘religion’ had been previously ranked as the primary motivator in the survey. Malaysians are ‘generally religious people’ <M_KL>, with approximately 62% identifying as Muslim [26], and government and societal regulations formulated in line with these beliefs. However, on questioning participants about the influence of religion on improving animal welfare it became clear that survey respondents may have been referring to the religious beliefs of their customers rather than their own beliefs, and to beliefs that are specifically about regulation and requirement. ‘It is more in regard to requirement, rather than belief, specifically in Malaysia [...] many top players (in agriculture) are not Muslim, but halal is state requirement’ <M_KL>. In this context, religion has become more akin to law, commanding a similar level of motivation. ‘Local government doesn’t just cover veterinary authorities, but also religious slaughter compliance [...] they can cease operations if (they) find you are not complying’ <M_KL>. ‘Instead of religious beliefs, I think it’s religious requirements. If you’re not required to do something you don’t’ <M_KL>. When asked if this was the case, and that ‘religion’ here almost refers to ‘law’, the participants in the Malaysia, Kuala Lumpur focus group agreed. This is logical; in Malaysia, religion, state and government are one, with the enactment of Sharia Law [28]. For this reason, religion is seen within this study as an extrinsic motivator and is presented as such in Figure 1.

## 11. Other: Financial Benefit

Financial benefit appeared as a consistent theme, with great emphasis placed on it by stakeholders in each of the four countries. This result is of particular extrinsic significance, as it was not rated highly in the previous survey study, and therefore was not specifically raised in any detail by the researcher in this focus group study. However, it was autonomously raised by participants throughout the sessions. 

As discussed in ‘company approval’ and ‘law’, the relationship of the financial gain as a motivator alongside law is clear. The law is perceived as being needed in the absence of company approval, which is given if financial benefit is perceived. If there is no law or monitoring in the market, the market will put more focus on production performance [...] and as we have also talked about, we do not have specific data to prove there is a positive connection between improvement of animal welfare and production performances we can give farmers’ <C_GZ>. ‘As a businessman I must be honest, when you have stakeholders to answer to, and talk about profit and loss at the same time as animal welfare, that’s why I ask, “what are the actual benefits?” When somebody wants to implement something new, you are answerable to expenses incurred. If you can’t justify that, you are in deep trouble’ <M_NS>.

Financial benefit primarily concerned consumer markets (domestic or export markets), drawing attention to the lack of consumer demand for higher welfare products in Asia. ‘I think that the customer may be more important than law, in (the) sense of the swine slaughterhouse’ <T_BK>. ‘The industry is the driving force of the promotion of animal welfare, and this comes from the demand of consumers and so that they can absorb demand of consumers and produce higher quality products’ <T_BK>. ‘If customers pay more, companies will provide’ <M_KL>. While there was a belief that export customers will pay more for higher welfare products ‘because the export customers demand higher welfare’ <T_KK>, the feeling was not necessarily similar when discussing domestic market opportunities. This was raised as a problem when international importers are demanding higher welfare animal products, for no increase in operating costs. ‘They want higher welfare standards but pay normal rate’ <T_BK>. The conflict of higher production and welfare was also raised, ‘Incentives to get more money if you get more production from the layer chickens <T_CM>. A price increase tied to the cost of improving animal welfare was also raised for the domestic market. ‘Sometimes if we have very small investment for better animal welfare, that means the customer is burdened with three or four times the price and it’s not fair’ <C_GZ>.

Financial benefit in China was perceived specifically in regard to offering consumers higher quality meat products, rather than higher welfare products. However, scepticism was expressed about whether the benefits of improved meat quality existed. ‘From a commercial perspective, I think the better way is to think of a plan to persuade customers good quality has come from better animal welfare [...] but I still think the quality of the meat is mainly decided by the breeds and nutrition’ <C_GZ>. 

At the core of the feedback from participants regarding financial benefit, was that animal agriculture was ultimately a business. ‘Cost factor needs to be considered as well. When additional cost (is) involved, companies need to review the cost; is it worth putting in the cost to get back the benefits?’ <M_KL>. ‘It’s a business at the end of the day. If a business doesn’t make the margin they need to, they don’t exist’ <M_KL>.

**Figure 2:**
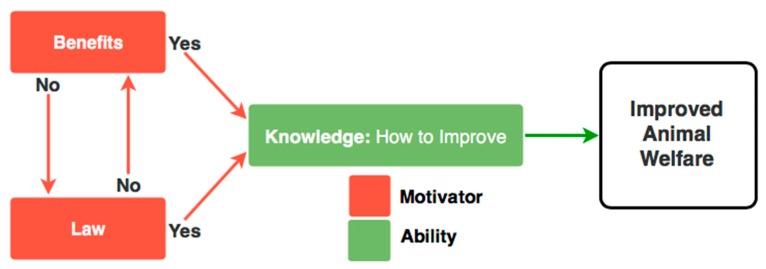
The relationship between law, and financial benefit as motivators to improve animal welfare in the Asian livestock industry.

The importance of financial benefit as a motivator was not reflected highly in the initial questionnaire study, probably because of the social bias tendency to underreport the importance of money as a motivator for action [29], and to report responses that are perceived to be more socially desirable. However, the focus group sessions allowed the facilitator to probe this further, and collect broader information, revealing that ‘financial benefit’ was of extreme importance as a motivator within the animal welfare landscape. In fact, money could be a stronger motivator than law and, importantly, potentially the strongest motivator where there is no law. Supported by appropriate knowledge, law and financial benefit are likely to result in action to improve animal welfare (see Figure 2). This revelation may have also been possible due to the focus group dynamic in which participants were able to deflect to ‘other’ stakeholders within the industry that they represent, rather than supply purely personal views, which were provided in the foundation survey study [25].

## 12. Other: Food Safety 

A further motivating factor that appeared in the data, despite not being asked in either the previous survey study or the current focus group study, was the need to provide safe food to consumers. Food safety was presented to the researcher as a concept that was well understood with good support, unlike animal welfare, and was suggested to provide opportunities for improving animal welfare standards uptake in all four of the countries, with particular focus on China. ‘I heard about how we (should) treat animals, but this is very new [...] we mostly do everything based on our habits/traditional methods, I heard about how to treat animals, but other issues are more important [...] food safety and disease control is more important’ <V_HN>. When one stakeholder in China stated, ‘the government is focused on food safety, so when improving food safety the animal welfare is being improved; there are no special funds from government on improving animal welfare, but could be tied to food safety [...] stakeholders in farms don’t know the words ‘animal welfare’ but (are) doing the job for improving food safety’ <C_ZZ>, it was met with passionate agreement from the group.

Where food safety was presented by participants in the focus group sessions, it was often tied again to profit. ‘If the farmers take care very well of the animals so they have no defects or any disease during production or transportation, then when they go to the slaughterhouse, the farmer will get higher money back’ <T_KK>.

‘Food safety and environmental protection are the two main things that the Chinese government focus on. China has focused on food safety and environmental for many years, but especially in recent years [...]. Now is a really good chance and time for proposal of animal welfare in China’ <C_ZZ>.

In the quantitative questionnaire precursor to this study, food safety was not presented as a motivator. For this reason, coupled with the smaller sample size in this study, as is usual in qualitative research as compared to quantitative, this motivator is not analysed against other motivators here. ‘Food safety’ as a theme will be analysed in full and presented qualitatively in a following publication.

## 13. Motivator Relationships and Application

The results of the primary quantitative questionnaire painted a useful initial description of the strength of the motivational forces on which to base this qualitative research, which aimed to gain further insight into motivators for changing animal welfare. Building on the preliminary results with a qualitative approach has revealed rich relationships of interwoven motivations, with deeper and more complex meaning, conditions and potential applications (see Figure 1). Within Figure 1, the two halves of the picture, ‘personal value’ (extrinsic or intrinsic) and ‘knowledge’ are both required to improve animal welfare. One without the other may be likely to result in inaction, or reduced welfare. The strength of each motivator (for e.g., money or religion) will vary with country, stakeholder role, and individual.

Figure 2 shows the perceived requirement of law to motivate stakeholders to improve animal welfare, in situations in which financial benefits are or are not present. Within Figure 2, the perceived requirement of law to motivate stakeholders to improve animal welfare, in situations in which financial benefits are or are not present. In each case, knowledge is required to empower stakeholders to bring about animal welfare improvement. This figure operates in the situation devoid of intrinsic motivation to improve animal welfare i.e., a desire to improve animal welfare based on internal values such as a sense of responsibility to the animals or a desire to reduce suffering.

Human motivation can be powered both intrinsically (internally, due to an interest or an ability to derive satisfaction from the task), or extrinsically (externally, due to an instrumentality that connects action with tangible external rewards) [30,31]. Intrinsic motivation is seen as ‘free choice’ [32], which in cases to do with animal welfare are underpinned by a belief in the value of the benefit to the animals and improvements themselves. While initiating intrinsic motivation would take longer where it isn’t already present, it would also arguably be the most compelling form of motivation for sustainability of actions to improve, in the absence of external pressures to do so. For the best results, intrinsic and extrinsic motivators would both be present [32], however, where intrinsic motivation does not exist, extrinsic motivators (such as law, company policy, financial benefit) can be very powerful, particularly when followed by value and knowledge (see Figure 1). 

It could be suggested that intrinsic motivations may be the most beneficial to long term and sustained action to improve animal welfare, demonstrated by the nature of comments and readily applied concern for animals often expressed by those participants most motivated intrinsically, but take a much longer time to instil than extrinsic motivations which can be applied more readily and strengthened with knowledge for positive outcomes in the short term. To this end, animal welfare initiatives would be best holistically focussed on appealing to both forms of motivation, with different aspects of initiatives created to develop the short term (mostly extrinsic) and long term (mostly intrinsic) motivations of stakeholders.

Although much research exists about human motivation theory in literature, very little exists in regard to the specific motivational forces/driving factors influencing the impetus to act. Less literature exists in this regard for animal welfare in a cross-border setting. For this reason, this work is foundational only, and will require a continued building of research and understanding of the complex cultural, political and economic landscape, specific to each of the study countries, and extrapolated from where the cultural profiles and agricultural, economic and political landscapes are similar. The findings of this study, combined with the precursor [14,25] and forthcoming studies, will provide an improved understanding of the stakeholders who should be a target of animal welfare initiatives, with the purpose of developing collaborative, rather than adversarial relationships built on mutual benefit [15]. This collaboration, built on mutual understanding and respect, is likely to result in improved animal welfare positions and practices.

## 14. Limitations

The primary limitation within this study resides within the process of translation, and that deep sentiments may not have been fully understood by the lead researcher/lead author due to an inability to speak each of the languages. For this reason, translators were briefed to deliver sentiments as true to participant intention as they were able. As a result of this, transcribing was not conducted word for word, and therefore tone and other communication subtleties were not recorded or analysed, and word counts were not presented. Steps were proactively taken within the sessions by the lead researcher to confirm that intentions and notions were captured accurately (consistent validation; summaries presented, amended and confirmed) with all participants. Despite aiming for a presence of 5–7 participants, requirements on the day meant some groups had less (as low as 3; Khon Khaen, Thailand), and enthusiasm to be involved in research meant other groups had more (as high as 14; Kuala Lumpur, Malaysia). In addition, group constitution was variable in terms of stakeholder roles within the industry. It was not apparent to the lead researcher that this altered group dynamic to the point that data was impacted, however this is a possibility for consideration. 

## 15. Conclusions

Based on motivating factors that were raised as statistically important in an earlier study [25], this qualitative focus group study revealed a complex picture of relationships between the identified motivators, and the contexts and conditions that drive them. Personal knowledge, as a motivational force to improve animal welfare, was discussed with the most frequency and emphasis by livestock stakeholders in this study. While this included the need for increased knowledge of animal welfare and agricultural processes amongst the general public, along with increased technical knowledge amongst livestock stakeholders themselves; this theme most importantly referenced the need for livestock stakeholder knowledge pertaining to the potential benefits to be gained by improving animal welfare. This knowledge was believed by participants to carry strong motivational value to improve animal welfare. The second most referenced theme within the study was that of law, as a motivational factor to improve animal welfare. Most commonly, stakeholders referenced the power and impact of the law, and its current strengths and gaps. The size and structure of the livestock company was thought to impact the motivational value of animal welfare law, with larger companies in a particularly advantageous position to take government issued law and directly implement into company policy, effectively giving their approval for animal welfare improvements to flow down throughout the supply chain. Company approval and policy, to many stakeholders, was seen to be just as important and sometimes synonymous with law, as a motivator to improve animal welfare. Motivating company approval, in the absence of law, was tied back to a knowledge of the potential benefits for improving animal welfare. Primarily, this can be taken to mean an equation to increased financial benefit. Financial benefit, as a motivator, was underreported in terms of importance in the earlier survey study. However, through the process of facilitating honest discussions in the present study, it emerged as a highly significant motivational factor for improving animal welfare, reported in all of the countries in this study. Food safety was also raised as an additional potential motivational factor for improving animal welfare in just over half of the sessions, despite not being presented to stakeholders as a finding of the previous survey study. This was particularly the case in China.

After analysis of these results, visual models are presented in Figure 1 and Figure 2 to assist understanding the motivational factors, and to better understand the livestock stakeholders at the core of animal welfare improvements. This paper also presents the opportunity to better understand the strength and relationship of extrinsic and intrinsic motivational forces behind animal welfare improvement. The findings and models presented in this study will provide further background information for those endeavouring to achieve higher animal welfare standards in this region.

## Figures and Tables

**Table 1:** Location of focus groups and abbreviation codes used in quote citations.

Country	Region	Abbreviated Code
Vietnam	Hanoi	V_HN
	Ban Me Thuot	V_BT
	Ho Chi Minh City	V_HC
Malaysia	Negeri Sembilan	M_NS
	Kuala Lumpur Selangor	M_KL
Thailand	Bangkok	T_BK
	Khon Kaen	T_KK
	Chiang Mai	T_CM
China	Guangzhou	C_GZ
	Zhengzhou	C_ZZ
	Beijing	C_BJ

**Table 2:** Summary of top five rankings factors influencing attitudes to animal welfare, from most important (top) to least important (bottom) by Chinese (n = 381), Thai (n = 307), Malaysian (n = 124) and Vietnamese (n = 210) respondents, as presented in Sinclair et al. in 2017 [25].

Discussion Points	China	Thailand	Malaysia	Viet Nam
‘The following factors influence my personal evaluation of animal welfare during slaughter and transport’	Laws ^a^ Knowledge ^ab^ Workplace ^ab^ More pressing concerns ^bc^ Co-workers ^cd^	Co-workers ^a^ Laws ^a^ Religious beliefs ^ab^ Knowledge ^abc^ Workplace ^bc^	Religious beliefs ^a^ Workplace ^b^ Knowledge ^b^ Laws ^b^ Co-workers ^b^	Laws ^a^ Knowledge ^a^ Workplace ^b^ Co-workers ^b^ More pressing concerns ^c^
‘The following factors impact my ability to make improvements to welfare during slaughter’	Laws ^a^ Tools and resources ^ab^ Company approval ^abc^ Knowledge ^bcd^ Work ^cde^	Laws ^a^ Company approval ^ab^ Co-workers ^ab^ Tools and resources ^ab^ Workspace ^ab^	Religious beliefs ^a^ Laws ^ab^ Knowledge ^ab^ Tools and resources ^ab^ Co-workers ^ab^	Laws^a^ Knowledge ^ab^ Co-workers ^ab^ Company approval ^abc^ Tools and resources ^bcd^
Sources of encouragement to make improvements to animal welfare	Law^a^ Personal value ^a^ Local government ^b^ Police ^bc^ Workplace ^bcd^ Local organization ^cde^	Personal value ^a^ Law ^ab^ Workplace ^ab^ Local organization ^bc^ Personal monetary gain ^bcd^ Local government ^bcde^	Law ^a^ Personal value ^ab^ Local government ^ab^ Workplace ^abc^ Local organization ^bc^ Supervisor ^cd^	Law ^a^ Personal value ^a^ Police ^b^ Local government ^b^ Workplace ^b^

Means that do not share a letter are significantly (*p* < 0.05) different by Fisher LSD test. Sinclair et. al. in 2017 [25]. Subscripts represent significance as per Fishers model of statistical analysis.

**Table 3:** Motivators (themes) and subthemes.

Theme	Sessions (Out of 11)	Referrals	Subtheme	Sessions (Out of 11)/References
Law	11	97	Need for standards	9/19
			Company size and structure	9/29
			Awareness of law	5/16
			Power and impact of law (strength and gaps)	10/30
Personal knowledge	11	185	General public knowledge of animal welfare	8/34
			Knowledge of benefits	10/16
			Stakeholders technical ability	7/25
Other—financial gain	10	71		
Personal value	10	43		
Tools and resources	10	31		
Workplace approval	7	21		
Other–food safety	6	14		
Religious beliefs	3	33		

Notes: ‘Themes’ indicate key motivators that were discussed within the sessions, with those commenced with ‘other’ indicating a motivator that was raised by the stakeholder participants, rather than as a product of previous results (Table 2). ‘Subthemes’ indicate a theme of ideas that were reflected with consistency in the context of each theme. ’Sessions’ quantifies the amount of stakeholder sessions (out of 11) that the theme or subtheme was presented with significance. ‘References’ quantifies the number of times the theme or subtheme was referred to in the data.

**Table 4:** Number of participants in each stakeholder group.

Country	Region	Stakeholder Group				Total
		Industry veterinarian	Industry business	Government	Academic	
Vietnam	Hanoi	4	1	2	0	7
	Ban Me Thuot	3	1	1	0	5
	Ho Chi Minh City	0	2	6	0	8
Malaysia	Kuala Lumpur Selangor	2	9	2	1	14
	Negeri Sembilan	2	1	3	1	7
Thailand	Bangkok	4	5	1	0	10
	Khon Kaen	0	2	1	0	3
	Chiang Mai	0	4	0	2	6
China	Guangzhou	0	2	0	5	7
	Zhengzhou	0	5	1	1	7
	Beijing	0	8	0	1	9
	**Total**	15	40	17	11	83
